# Polio: The Disease that Reemerged after Six Years in Ethiopia

**DOI:** 10.4314/ejhs.v31i4.25

**Published:** 2021-07

**Authors:** Sintayehu Tsegaye Tseha

**Affiliations:** 1 Infection biology PhD candidate at the department of microbial, cellular and molecular biology, Addis Ababa University, Addis Ababa, Ethiopia; MSc in Biomedical sciences, Lecturer of Biomedical Sciences at Arba Minch University

**Keywords:** Polio, Ethiopia, Poliovirus, reemergence, circulating vaccine-derived poliovirus

## Abstract

**Background:**

Polio is a disabling and potentially deadly disease caused by a wild poliovirus and vaccine-derived poliovirus. The purpose of this review is to discuss the current situation of polio in Ethiopia.

**Method:**

Relevant scientific articles on Polio were searched from different data bases and websites.

**Results:**

The first wild poliovirus in Ethiopia was detected in 1999, followed by detection of few cases in 2000 and 2001. No wild poliovirus was detected in Ethiopia for the next 3 years (2001–2003). However, the disease resurged again in the country between 2004 and 2008 due to challenge to provide sufficient oral poliovirus vaccine coverage, migration and cross border economic activities and lack of good acute flaccid paralysis surveillance. After almost 5 years with no wild polio virus, Ethiopia again affected by polio outbreak importation in 2013. However, due to multiple supplementary immunization activities campaigns of improved quality and enhanced surveillance, the outbreak was eventually successfully interrupted within 6 months of confirmation. The most recent emergence of polio in Ethiopia has seen in this year (2020) six years after the country documented zero polio cases since 2014. The cause of the resurgence of the disease is circulating vaccine derived polio virus-2. Currently, Ethiopia has been conducting outbreak response by declaring Mop-up campaigns since September 2020.

**Conclusions:**

Therefore, it can be recommended that: - 1. The country has to completely shift from oral polio virus vaccine to inactivated polio vaccine so that the risk of vaccine derived polio will be diminished; 2. Ethiopia has to strengthen the mop up campaign that it has started in September 2020 following the reemergence of the disease in the country; 3. Ethiopia has to strengthen surveillance for acute flaccid paralysis in order to rapidly detect any new virus importation and to facilitate a rapid response.

## Introduction

Polio (poliomyelitis) is a disabling and potentially deadly disease caused by a wild poliovirus and vaccine-derived poliovirus (1). Before the Global Polio Eradication Initiative (GPEI) started, polio paralyzed more than 1000 children worldwide every day (2). Since the establishment of the Global Polio Eradication Initiative in 1988, there has been considerable reduction in polio cases from more than 350,000 cases in 1988 to 33 cases in 2018 (3,4). However, the global situation remains of great concern with the increased number of polio cases that started in 2019 continuing in 2020. This year there have been 70 wild poliovirus type 1 (WPV1) cases as of 16 June 2020, compared to 57 for the same period in 2019, with no significant success yet in reversing this upward trend (5). In this year, in Ethiopia, the disease has been seen in some parts of the country (5). The purpose of this review is to discuss polio situation in Ethiopia.

## Epidemiology of Polio

Polio is a disease of only man. It is very contagious and spreads from person to person mainly through contact with feces of infected person and rarely by contaminated water or food (4). In the early 20th century, polio was one of the most feared diseases in the world, including industrialized countries, paralyzing hundreds of thousands of children every year (2). Before the Global Polio Eradication Initiative (GPEI) started, polio paralyzed more than 1000 children worldwide every day (2).

In 1988, the World Health Assembly established the Global Polio Eradication Initiative (GPEI) with goal to eradicate polio from the world, spearheaded by national governments, World Health Organization (WHO), Rotary International, the US Centers for Disease Control and Prevention (CDC), United Nations Children's Fund (UNICEF), and the Bill & Melinda Gates Foundation and the global Vaccine Alliance for vaccine and immunization (GAVI) (6). Since then, more than 2.5 billion children have been immunized against polio thanks to the cooperation of more than 200 countries and 20 million volunteers (2). As a result, Wild poliovirus cases have decreased by over 99% in 2018 (4).

However, today the disease is remerged in several countries in the world. According to WHO, currently, the disease is now detected in four WHO regions (African, Eastern Mediterranean, South-east Asian and Western Pacific Regions) (5). This is due to cVDPV2 and COVID-19 pandemic which adversely affected routine immunization of polio vaccine (5).

Poliomyelitis affects all age groups. But the disease is commonly seen in nonimmunized individuals, but only about 1 percent of these cases progress to the paralytic form of the disease. The most susceptible are children less than 2 years old (4). In countries which have high poliomyelitis immunization coverage in their childhood vaccination programs there is a shift to an older age group. Poor sanitation, inadequate water supply and crowded living conditions were reported as risk factors for communities highly affected by the disease (7).

There has been success in eradicating two of the three strains of the virus: the last case of type 2 was reported in 1999 and its eradication was declared in September 2015. The most recent case of type 3 dates to November 2012 in Nigeria and this strain was declared as globally eradicated in October 2019 (3, 4). Today, wild poliovirus type 1 affects only two countries: Pakistan and Afghanistan (8).

## Structure and Taxonomy of Poliovirus

Poliovirus belongs to the family *Picornaviridae* (9,10). Viruses in the family *Picornaviridae* are small, non-enveloped viruses (size ranging from 22 to 30 nm) with icosahedral capsid symmetry and has RNA genome. Poliovirus has singlestranded positive-sense RNA genome that is about 7500 nucleotides long (11). Because of its short genome and its simple composition (only consisted of RNA and a protein coat that encapsulates it, poliovirus is widely regarded as the simplest significant virus (12). Poliovirus has three serotypes: wild poliovirus type 1(WPV1), wild poliovirus type 2 (WPV2), wild poliovirus type 3 (WPV3). All of the three serotypes can cause irreversible paralysis or even death (13). However, the capsid proteins of each of the three serotypes of poliovirus are slightly different, which defines their cellular receptor specificity and their antigenicity. WPV-1 is the most common form encountered in nature, but all three forms are extremely infectious (14).

## Clinical Features and Pathogenesis of Polio

Poliovirus has tropism for epithelial cells of the alimentary tract and cells of the central nervous system and viral replication occurs in the alimentary tract and neurons (10). Poliovirus infects host cells by binding its ligand with its immunoglobulin-like receptor, which is called CD155, that is also known as the poliovirus receptor (PVR) (15–17). The binding of the virus with its receptor (CD155) results in a conformational change of the viral particle necessary for viral entry (18,19). Following attachment to the host cell membrane, entry of the viral nucleic acid was hypothesized to occur in one of the following two ways: either via the formation of a pore in the plasma membrane through which the RNA is then injected into the host cell cytoplasm, or via virus uptake by receptor-mediated endocytosis (20).

The incubation period is about 3 to 5 days for minor illness and 1 to 2 weeks for central nervous system involvement (10). Replication of the virus has four phases: the alimentary phase, lymphatic phase, the viremic phase and neurologic phase. Primary replication of the virus takes place in the intestinal and the oropharyngeal mucosa, which is called the alimentary phase. Then, the virus spreads to the tonsils and Peyers patches and to deep cervical and mesenteric nodes, where it multiplies abundantly (the lymphatic phase). Following this, the virus is transported by the bloodstream to various internal organs and regional lymph nodes (the viremic phase) (10).

Sometimes, the virus enters the central nervous system (CNS) and replicates in motor neurons within the spinal cord, brain stem, or motor cortex, resulting in the selective destruction of motor neurons leading to temporary or permanent paralysis, a phase called the neurologic phase (paralytic disease). Paralytic disease is a very rare event in babies who have anti-poliovirus antibodies acquired from their mothers (21). Paralytic poliomyelitis may rarely bring about respiratory arrest and death. Muscle pain and spasms are frequently observed prior to onset of weakness and paralysis in cases of paralytic disease. Paralysis usually continues from days to weeks prior recovery (22). Paralytic disease had two forms: 1. Spinal poliomyelitis and bulbar poliomyelitis. Spinal poliomyelitis is weakness that is limited to muscles innervated by the motor neurons in the spinal cord, whereas, the bulbar poliomyelitis refers to weakness that is limited to muscles innervated by the motor neurons in the cranial nerve nuclei or medullary centers (10).

Factors that influence the outcome of poliovirus infection include: 1. The virulence of the infecting strain, the immune status of the host and the size of the infecting dose of the virus. Children under five years, pregnant woman, those with tonsillectomy and immunocompromised persons are predisposed to more severe illness (10).

In 95% of cases, the poliovirus infection is asymptomatic (23). Paralytic disease (temporary or permanent acute flaccid paralysis (AFP), occurs in less than 1% of polio the cases (23). Nearly, half of all survivors of acute poliomyelitis will experience post-poliomyelitis syndrome (PPS) (24), being the delayed appearance of new or worsening disabling neuromuscular symptoms 30–40 years after the first poliomyelitis attack. 5% to 10% of those with paralytic disease die when their breathing muscles become immobilized (4). Only about 1 in 10 people recover from paralyzed polio cases (25).

## Prevention and Control of Polio

Laboratory diagnosis of the disease is based on serology. There is no specific therapy for poliovirus (10). Poliomyelitis can be prevented by vaccine. There are two types of polio vaccine Salk-type (inactivated polio vaccine, IPV) and Sabin-type (live attenuated oral poliovirus vaccine (OPV). Prevention can also be achieved via public education on transmission modes and personal hygiene. Adequate sewage disposal and uncontaminated water supplies are critical for prevention of poliovirus infection.

Polio vaccine has been available since 1955 (25). The Salk-type (IPV) is the first polio vaccine, which is given by intramuscular injections spaced a month apart and requires periodic boosters to maintain an adequate serum neutralizing-antibody level. The IPV has suppressive effect on wild poliovirus replication in the highly vascularized oropharyngeal region. However, the Salk-type has no effect on replication in the gut (10).

The Sabin-type (live attenuated oral poliovirus vaccine) (OPV) was developed in 1961, which is given orally, dropped into mouth (25). This vaccine mimics wild poliovirus infections by inducing serum-neutralizing antibody, as well as interferon and virus-specific IgA antibody in the pharynx and gut (10). When epidemic occurs, trivalent OPV is recommended. However, just after identifying the responsible poliovirus serotype, monovalent OPV containing the causative serotype have be administered as soon as possible. The OPV is effective in preventing the disease which has been shown to prevent a threatening epidemic and has the potential of eradicating poliomyelitis. However, it is not without draw back. The major disadvantage of OPV is the occurrence of vaccine-associated paralysis (25). Recently, a combination of IPV and OPV vaccination strategy used in the control and eradication of poliomyelitis in most parts of the world (26).

Polio is one of few types of diseases that can be eradicated. Polio can be eradicated from the world because of the following reasons (27): 1. The poliovirus infects only humans 2. There is effective and cheap vaccine; 3. Immunity against polio is life-long and the virus can only survive for a very short time in the environment. The following four are the strategies that have been used within the Polio Eradication Initiative i) routine immunization, ii) supplementary immunization activities (SIAs), iii) disease surveillance and iv) mop-up campaigns.

## Polio Situation in Ethiopia

In Ethiopia, wild poliovirus was first detected in 1999. Following this, three cases due to wild poliovirus were detected in 2000 and 2001. No wild poliovirus has been detected in Ethiopia for the last 3 years (2001–2003) (28). This considerable success is due to the fact that Ethiopia has achieved tremendous progress in its Polio Eradication Initiative activities since it commended in 1996. OPV coverage rates have increased appreciably (from less than 400,000 children in 1996 to more than 14 million in 2001) leading to reduced transmission of the virus (27).

However, the disease remerged between 2004 and 2008 in Ethiopia due to combination of factors, which include: - challenge to provide sufficient OPV coverage, migration, cross border economic activities and lack of sufficient AFP surveillance in different parts of the country (27). Ethiopia had five WPV importations between 2004 and 2008 (29). In 2004, the disease was detected in northern part of the country, which was imported from Nigeria through Chad and Sudan. The 2005 and 2006 wild polioviruses were shown to be genetically linked to the viruses circulating in Sudan and Somalia, respectively. The importation in Gambella region in 2008 was genetically linked to the virus circulating in southern Sudan (30–33).

After almost 5 years with no wild polio virus, Ethiopia again affected by polio outbreak importation in 2013, when Horn of Africa (HOA) countries experienced more outbreak associated cases of WPV type 1 (223 cases) than had ever been documented before, which affected Somalia, Kenya, and Ethiopia (34, 35), which was closely linked to the poliovirus circulating in Nigeria. However, due to multiple supplementary immunization activities (SIAs) campaigns of improved quality and enhanced surveillance (29), the outbreak was eventually successfully interrupted within 6 months of confirmation.

Today, re-emergence of polio has occurred in Ethiopia after six years after the country documented zero polio cases since 2014(5). According to WHO, cVDPV2 was suggested as the reason for the resurgence of the disease in Ethiopia (5). Currently, Ethiopia has been conducting outbreak response by declaring Mopup campaigns since September 2020.

In summary, Polio has reemerged after six years in Ethiopia. cVDPV2 is the cause for the resurgence of the disease in Ethiopia. It can be recommended that: -1. In spite of its expensiveness and difficulty in administration relative to OPV, Ethiopia has to completely shift from OPV to IPV as USA did in 2020 so as to eliminate the disease from the country by avoiding the risk of vaccine derived polio that may arise from OPV; 2. Ethiopia has to strengthen the mop up campaign that has been started in September 2020 just after the reemergence of the disease in the country; 3. Ethiopia has to strengthen surveillance for AFP in order to rapidly detect any new virus importation and to facilitate a rapid response and 4. Ethiopia has to take the necessary measures to avoid importation of the virus particularly from polio affected countries.
